# Accelerating Discovery of Leukemia Inhibitors Using AI-Driven Quantitative Structure-Activity Relationship: Algorithm Development and Validation

**DOI:** 10.2196/81552

**Published:** 2026-01-27

**Authors:** Samuel Kakraba, Edmund Fosu Agyemang, Robert J Shmookler Reis

**Affiliations:** 1 Biostatistics and Data Science Celia Scott Weatherhead School of Public Health and Tropical Medicince Tulane University New Orleans, LA United States; 2 Tulane School of Medicine Tulane Center for Aging Tulane University New Orleans, LA United States; 3 Department of Geriatrics School of Medicine University of Arkansas for Medical Sciences Little Rock, AR United States

**Keywords:** anti-leukemia, thiadiazolidinones, TDZD analogs, artificial intelligence, machine learning, quantitative structure-activity relationship, QSAR, small-molecule inhibitors, drug discovery, precision oncology, Shapley additive explanations analysis

## Abstract

**Background:**

Leukemia treatment remains a major challenge in oncology. While thiadiazolidinone analogs show potential to inhibit leukemia cell proliferation, they often lack sufficient potency and selectivity. Traditional drug discovery struggles to efficiently explore the vast chemical landscape, highlighting the need for innovative computational strategies. Machine learning (ML)–enhanced quantitative structure-activity relationship (QSAR) modeling offers a promising route to identify and optimize inhibitors with improved activity and specificity.

**Objective:**

We aimed to develop and validate an integrated ML-enhanced QSAR modeling workflow for the rational design and prediction of thiadiazolidinone analogs with improved antileukemia activity by systematically evaluating molecular descriptors and algorithmic approaches to identify key determinants of potency and guide future inhibitor optimization.

**Methods:**

We analyzed 35 thiadiazolidinone derivatives with confirmed antileukemia activity, removing outliers for data quality. Using Schrödinger MAESTRO, we calculated 220 molecular descriptors (1D-4D). Seventeen ML models, including random forests, XGBoost, and neural networks, were trained on 70% of the data and tested on 30%, using stratified random sampling. Model performance was assessed with 12 metrics, including mean squared error (MSE), coefficient of determination (explained variance; *R*^2^), and Shapley additive explanations (SHAP) values, and optimized via hyperparameter tuning and 5-fold cross-validation. Additional analyses, including train-test gap assessment, comparison to baseline linear models, and cross-validation stability analysis, were performed to assess genuine learning rather than overfitting.

**Results:**

Isotonic regression ranked first with the lowest test MSE (0.00031 ± 0.00009), outperforming baseline models by over 15% in explained variance. Ensemble methods, especially LightGBM and random forest, also showed superior predictive performance (LightGBM: MSE=0.00063 ± 0.00012; *R*^2^=0.9709 ± 0.0084). Training-to-test performance degradation of LightGBM was modest (Δ*R*^2^=–0.01, ΔMSE=+0.000126), suggesting genuine pattern learning rather than memorization. SHAP analysis revealed that the most influential features contributing to antileukemia activity were global molecular shape (*r_qp_glob*; mean SHAP value=0.52), weighted polar surface area (*r_qp_WPSA*; ≈0.50), polarizability (*r_qp_QPpolrz*; ≈0.49), partition coefficient (*r_qp_QPlogPC16*; ≈0.48), solvent-accessible surface area (*r_qp_SASA*; ≈0.48), hydrogen bond donor count (*r_qp_donorHB*; ≈0.48), and the sum of topological distances between oxygen and chlorine atoms (*i_desc_Sum_of_topological_distances_between_O.Cl*; ≈0.47). These features highlight the importance of steric complementarity and the 3D arrangement of functional groups. Aqueous solubility (*r_qp_QPlogS*; ≈0.47) and hydrogen bond acceptor count (*r_qp_accptHB*; ≈0.44) were also among the top 10 features. The significance of these descriptors was consistent across multiple algorithmic models, including random forest, XGBoost, and partial least squares approaches.

**Conclusions:**

Integrating advanced ML with QSAR modeling enables systematic analysis of structure-activity relationships in thiadiazolidinone analogs on this dataset. While ensemble methods capture complex patterns with high internal validation metrics, external validation on independent compounds and prospective experimental testing are essential before broad therapeutic claims can be made. This work provides a methodological foundation and identifies molecular features for future validation efforts.

## Introduction

Leukemia remains a formidable challenge in oncology, largely due to the persistence of leukemia stem cells (LSCs), which drive disease relapse through intrinsic resistance to conventional chemotherapy [1]. While standard treatments effectively target proliferating leukemic blast cells, LSCs evade destruction by leveraging quiescence and enhanced survival mechanisms, such as dysregulated kinase signaling and adaptation to oxidative stress [1]. Thiadiazolidinone analogs, notably thiadiazolidinone-8, comprise a promising family of molecules that selectively induce rapid cell death in LSCs via a dual mechanism: (1) inhibition of glycogen synthase kinase 3β (GSK3β), and (2) triggering oxidative collapse [1]. Molecular docking and simulation studies suggest that thiadiazolidinone-8 might bind to an allosteric hydrophobic pocket in GSK3β’s inactive “DFG-out” conformation, preventing reactivation and disrupting prosurvival pathways, while simultaneously depleting intracellular thiols to disrupt membrane integrity within 2 hours, achieving 85% to 93% lethality in primary acute myeloid leukemia, acute lymphoblastic leukemia, and chronic lymphoblastic leukemia specimens at 20 μM. Critically, thiadiazolidinone-8 spares normal hematopoietic stem cells (79.5% viability) and significantly reduces engraftment of leukemic cells in nonobese diabetic/severe combined immunodeficient xenotransplantation models, with mean engraftment dropping from 76% to as low as 0.7% (*P*<.001), while having minimal toxicity for normal cells [1]. Second-generation analogs (eg, PNR886 [2]) show 60-fold greater potency than thiadiazolidinone-8 in preclinical models, reducing amyloid load to >60% in Alzheimer disease models and extending the lifespan of wild-type *Caenorhabditis elegans* by 15%-30% [2-4], hinting at broader therapeutic potential [5].

Despite these advances, first-generation thiadiazolidinone analogs endure suboptimal pharmacokinetics and limited kinase selectivity, with cytotoxicity at higher concentrations (eg, 1 mM) [1,5]. Recent computational modeling of GSK3β’s inactive state offers opportunities for the rational design of next-generation inhibitors targeting key residues (Lys205, Asp200, and Ala204) to enhance specificity and reduce off-target effects on normal tissues [5]. Structural optimization is essential to balance potent LSC eradication with minimal toxicity, unlocking the potential of thiadiazolidinone-based therapies to target the LSC reservoir in refractory leukemias specifically.

The quest for effective leukemia inhibitors is hindered by challenges such as enzyme specificity, cell selection for resistance, and off-target effects. Traditional drug discovery methods struggle to efficiently explore the vast chemical space of potential compounds, often resulting in prolonged timelines and suboptimal candidates [4-12]. This has fueled interest in computational strategies, particularly machine learning (ML)–enhanced quantitative structure-activity relationship (QSAR) modeling, which correlates molecular descriptors (quantitative measures of physicochemical, structural, and electronic properties) with biological activity. ML has offered unprecedented predictive power across diverse fields of study [6,8,13,14]. Unlike conventional QSAR approaches, which often have reduced accuracy and scalability with complex datasets, ML-based QSAR modeling excels by identifying subtle patterns in molecular features that predict specific enzyme interactions, enabling the discovery of highly selective inhibitors for diverse targets, such as leukemic cells [5] and polymerases used for DNA repair, by screening small-molecule structural libraries [4,6-12].

ML algorithms have shown promise in enhancing drug discovery [4,9,13-15] by enabling prediction of resistance mechanisms, guiding the design of inhibitors to delay or overcome resistance, and prioritizing molecular features linked to selectivity or minimal toxicity [5]. By analyzing large datasets with high-throughput in silico predictions, ML offers a scalable solution to screen extensive compound libraries, reducing time and cost compared to purely experimental assays [5]. Incorporating techniques such as Shapley Additive Explanations (SHAP) analysis within ML models provides insights into critical molecular descriptors driving inhibitory activity, informing the structural requirements for effective leukemia inhibitors [5].

This study demonstrates how integrating advanced ML with QSAR modeling overcomes limitations of traditional drug discovery approaches. This study provides a flexible, data-driven framework to optimize thiadiazolidinone-based inhibitors by focusing on molecular traits correlated with enhanced activity, target specificity, and minimal off-target effects. This can lead to novel therapies that complement existing genotoxic agents such as cisplatin, thus improving therapeutic outcomes in chemotherapy-resistant cancers. However, we acknowledge that such potential can only be realized through rigorous external validation and experimental verification of computational predictions.

## Methods

### Methodology for Enhanced Inhibitor Identification

We introduce a structured methodology to enhance the identification of thiadiazolidinone analogs with antileukemic properties using artificial intelligence (AI)–powered QSAR modeling. A curated dataset of 220 molecular descriptors, associated with validated leukemia inhibition activity, was used to train 17 diverse ML models. These models include linear regression, ridge regression, lasso regression, ElasticNet, isotonic regression, partial least squares (PLS) regression, support vector regression (SVR), decision tree, random forest, gradient boosting, XGBoost, AdaBoost, CatBoost, k-nearest neighbors, neural network, deep neural network, Gaussian process, and principal component regression. Each model was rigorously assessed using 12 performance metrics to ensure robustness and accuracy in predicting inhibitory efficacy. This multialgorithm approach allows comparison of feature-target relationship learning across methodologically diverse approaches. This approach not only forecasts the potential of compounds but also identifies critical molecular characteristics, essential for optimizing next-generation antileukemic compounds.

### Dataset and Preprocessing

#### Overview

##### Multistep Protocol

This study used an in-house selected library of 35 thiadiazolidinone analogs, each with experimentally validated leukemia inhibition activity expressed as *logIC_50_* values [1].

Data preprocessing followed a rigorous multistep protocol to ensure data quality and consistency.

##### Outlier Detection and Removal

Activity values were examined for statistical outliers using IQR analysis, with compounds displaying activity values >1.5×IQR from the quartile boundaries flagged for review and removed if deemed measurement anomalies.

##### Chemical Structure Standardization

Chemical structures were initially sketched in ChemDraw [16], converted to Simplified Molecular Input Line Entry System format, and subsequently transformed into SYBYL Mol2 files using Schrödinger MAESTRO (Schrödinger Release 2025-2: Canvas, Schrödinger, LLC, 2025) for 3D visualization, ensuring standardized chemical representation across all compounds.

##### Ligand Geometric Optimization

Ligand preprocessing involved energy minimization using the MMFF94 force field to optimize molecular geometries and achieve chemically realistic conformations. Structural alignment of conserved thiadiazolidinone cores was performed to standardize side-chain modifications across the dataset, ensuring consistent and comparable descriptor computation [17].

##### Descriptor Calculation

Molecular descriptors were calculated using Schrödinger MAESTRO 12.5 software, encompassing a broad spectrum of physicochemical properties (1D-4D descriptors). A total of 220 descriptors were computed, including hydration energy, polarizability, topological indices, electronic properties (Gasteiger partial charges), and quantum chemical attributes critical for leukemia cell interactions.

##### Feature Scaling and Normalization

Before model training, all molecular descriptor features were normalized using StandardScaler (*z* score normalization: (x – mean)/SD) to ensure equal weighting across features with different scales and units, preventing high-magnitude descriptors from dominating the learning process.

##### Missing Value Handling

Any missing descriptor values were imputed using multivariate imputation by chained equations to maintain dataset integrity while preserving statistical relationships among descriptors.

The resulting preprocessed dataset contained 35 compounds with 220 standardized molecular descriptors and corresponding experimental *logIC_50_* values, forming a robust foundation for QSAR modeling (see Multimedia Appendix 1 for the complete molecular database of molecular descriptors with corresponding *logIC_50_*).

### Model Training and Evaluation

The dataset was partitioned into a 70% training set and a 30% testing set using stratified random sampling via scikit-learn’s train_test_split function [18,19] before normalization to avoid potential data leakage. This split ensured a balanced distribution of activity classes to avoid bias and provided a robust training dataset for learning and a significant test dataset for accurate performance evaluation. Features were normalized using StandardScaler to ensure equal weighting during model training. The 17 ML algorithms evaluated spanned a wide range of approaches, including linear models, tree-based ensembles, kernel methods, instance-based learners, neural networks, probabilistic approaches, dimensionality reduction techniques, nonparametric models, and advanced gradient boosting frameworks. Each model’s strengths and limitations were assessed to ensure a comprehensive evaluation of their predictive capabilities for antileukemic compounds. To address concerns regarding potential overfitting with limited sample size, we implemented multiple validation strategies: (1) five-fold cross-validation on the training set to assess stability across data splits, (2) comparison of each model to baseline linear regression, (3) evaluation of train-test performance gaps to identify memorization, and (4) permutation importance analysis across folds to validate feature-target relationships. Performance metrics such as coefficient of determination (explained variance; *R*^2^), root-mean-square error in prediction, and others were used to quantify predictive accuracy and model robustness.

### Overview of ML Algorithms

The 17 ML algorithms compared for QSAR modeling are summarized in Table 1, detailing their descriptions, strengths, and limitations. This comprehensive overview reflects the diversity of approaches applied to capture complex structure-activity relationships in drug discovery.

**Table 1:** Overview of machine learning algorithms compared for QSAR^a^ modeling [20].

Algorithm	Description	Strengths	Limitations	References
Linear regression	Models relationships with a linear equation	Simple, efficient, highly interpretable	Assumes linearity, sensitive to outliers	[21]
Ridge regression	Uses L2^b^ regularization to prevent overfitting of data	Improves stability and handles multicollinearity	Does not perform feature selection	[22,23]
Lasso regression	Applies L1^c^ regularization for feature selection	Reduces model complexity through feature selection	May arbitrarily select among correlated variables	[24,25]
ElasticNet	Combines L1 and L2 regularization	Balances the benefits of lasso and ridge	Requires tuning 2 hyperparameters	[22,23]
Isotonic regression	Fits a monotonic free-form line to the data	Robust to outliers, ensures monotonic relationships	Computationally intensive, limited generalization	[26,27]
PLS^d^	Identifies relationships between matrices, reducing dimensionality	Manages multicollinearity, effective for high-dimensional data	Less interpretable than other methods	[28-30]
SVR^e^	Approximates input-output in high-dimensional space	Robust against data overfitting, excels in complex datasets	Sensitive to kernel choice, computationally intensive	[31-33]
Decision tree	Nonparametric tree structure for regression or classification	Interpretable, handles diverse data, and captures nonlinearity	Prone to overfitting, may not generalize well	[13,14,34,35]
Random forest	Ensemble of trees to minimize overfitting	Reduces overfitting, assesses feature importance	Computationally expensive, less interpretable	[13,14,34,36,37]
Gradient boosting	Builds weak learners sequentially for improved predictions	High predictive power, excels in complex modeling	Risk of overfitting if not tuned properly	[38,39]
XGBoost	Optimized gradient boosting library for enhanced performance	High accuracy, efficient, and handles missing data	Complex to tune, less interpretable	[40]
AdaBoost	Combines weak classifiers, focusing on misclassified instances	Improves accuracy by emphasizing difficult cases	Sensitive to noisy data and outliers	[41,42]
CatBoost	Uses ordered boosting for categorical features	Reduces overfitting, high accuracy with categorical data	Slower training speed, less interpretable	[43,44]
KNN^f^	Nonparametric method based on proximity to nearest points	Captures complex relationships without assumptions	Computationally intensive, sensitive to scaling	[45,46]
Neural network	Mimics brain processes to model nonlinear relationships	Adaptable, excels with large datasets	Requires significant data, prone to overfitting	[13,14,34,47,48]
DNN^g^	Advanced neural network with multiple layers for complex patterns	High performance in capturing intricate patterns	Requires large datasets, computationally intensive	[49,50]
Gaussian process	Probabilistic approach with uncertainty estimates	Offers uncertainty quantification, models complex functions	Computationally expensive for large datasets	[51]
PCR^h^	Combines PCA^i^ with regression for dimensionality reduction	Handles multicollinearity, reduces dimensionality	May lose interpretability, less predictive power	[52-54]

^a^QSAR: quantitative structure-activity relationship.

^b^L2: ridge penalty

^c^L1: lasso penalty

^d^PLS: partial least squares.

^e^SVR: support vector regression.

^f^KNN: k-nearest neighbors.

^g^DNN: deep neural network.

^h^PCR: principal component regression.

^i^PCA: principal component analysis.

Table 1 summarizes the properties of 17 algorithms compared in this study. The results were consistent with recent advances in QSAR modeling in which ML techniques such as random forest, XGBoost, and deep neural network empirically displayed superior predictive performance, especially for complex and diverse datasets [34]. The selection of these algorithms was guided by their established effectiveness in small-sample, high-dimensional biological datasets, their ability to handle multicollinearity, capture nonlinear relationships, and to provide insights into feature importance [34], all of which are critical for optimizing thiadiazolidinone-based inhibitors in leukemia treatment.

Hyperparameters were optimized via grid or random search with 5-fold cross-validation, prioritizing the minimization of mean squared error (MSE) and maximization of *R*^2^ and adjusted coefficient of determination (adjusted *R*^2^) metrics.

Model performance was evaluated using 12 metrics, including MSE, root-mean-squared error (RMSE), mean absolute error (MAE), mean absolute percentage error (MAPE), symmetric mean absolute percentage error (SMAPE), median absolute error (MedAE), *R*^2^, adjusted *R*^2^, concordance correlation coefficient (CCC), normalized mean squared error (NMSE), normalized root-mean-squared error (NRMSE), and Pearson correlation to ensure a comprehensive assessment of predictive accuracy and robustness. Detailed descriptions of these metrics are in the following sections.

### About MSE

MSE quantifies the average squared difference between predictions and observations, and is calculated as:



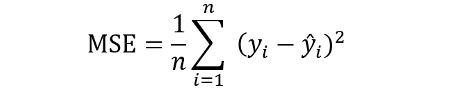



where *y*_i_ is the observed value and 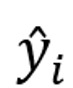 is the predicted value. MSE is critical for identifying models prone to severe inaccuracies.

### About RMSE

RMSE provides error magnitude in the same units as the response variable, enhancing interpretability and sensitivity to outliers. It is calculated as:



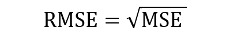



### About MAE

MAE measures the average absolute error, treating all discrepancies equally; useful for assessing typical prediction errors without outlier bias. It is calculated as:



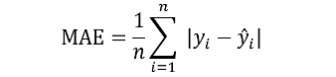



### About MAPE

MAPE expresses errors as percentages, facilitating relative performance comparison across datasets, though it is undefined for 0 observed values. It is calculated as:



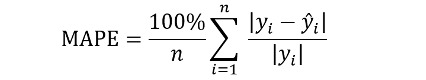



### About SMAPE

SMAPE addresses MAPE’s asymmetry by normalizing errors against the average of observed and predicted values, improving robustness for near-zero values. It is calculated as:



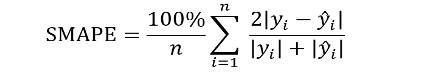



### About MedAE

MedAE is resistant to outliers and is calculated as:



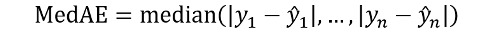



### About *R*^2^

*R*^2^ represents the proportion of variance explained by the model, with values closer to 1 indicating a better fit. It is calculated as:



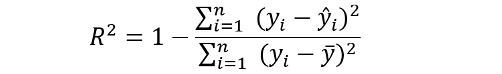



where 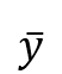 is the mean of observed values.

### About Adjusted *R*^2^

*R*^2^ adjusts for model complexity, preventing overfitting by penalizing unnecessary predictors. It is calculated as:



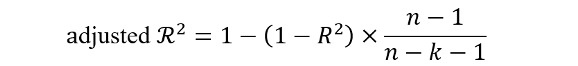



where:

- *R*^2^=*R*^2^ of the model, also known as the fraction of variance explained.
- n=number of observations (data points).
- k=number of predictors (independent variables) in the model.

### About CCC

CCC evaluates agreement between predictions and observations, combining precision (correlation) and accuracy (mean shift). It is calculated as:



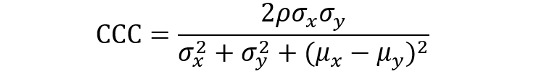



where *ρ* is Pearson correlation, and *μ* and *σ* are means and SDs of the observed and predicted values, respectively.

### About NMSE

NMSE scales MSE by dataset variance, enabling cross-study comparisons. It is calculated as:



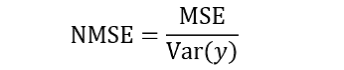



### About NRMSE

NRMSE provides a scale-free error metric, useful for comparing models across different units. It is calculated as:



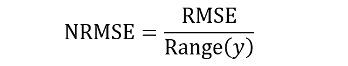



where:

range(y) = max(y) – min(y)

### Pearson Correlation Coefficient

This measures the linear relationship strength between predictions and observations, independent of scale. It is calculated as:



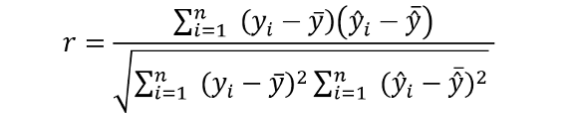



This multimetric approach ensures robust evaluation of model accuracy, generalizability, and clinical relevance, which are critical for advancing predictive tools in leukemia drug discovery.

**Figure 1:**
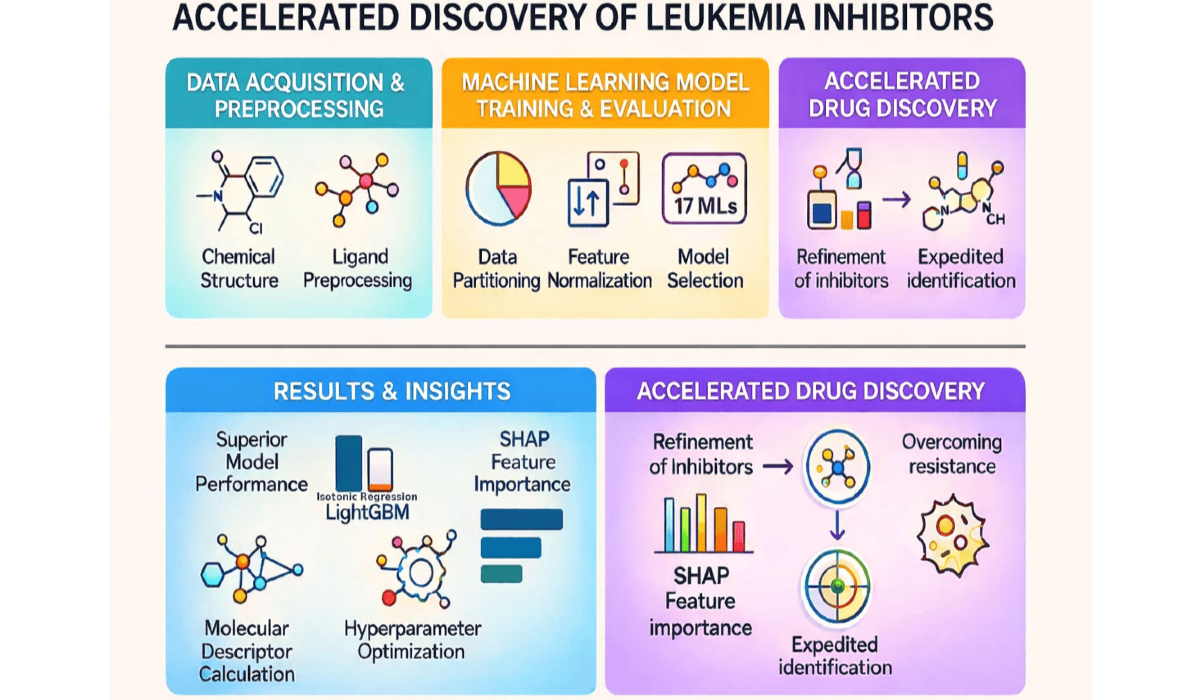
Graphical abstract depicting the integrated computational workflow for systematic analysis of structure-activity relationships in thiadiazolidinone analogs using machine learning-enhanced QSAR modeling. ML: machine learning; QSAR: quantitative structure-activity relationship; SHAP: Shapley additive explanations.

Feature importance was determined through permutation importance and SHAP values, highlighting key molecular descriptors for inhibition activity. Permutation importance was evaluated across all 5 cross-validation folds to assess consistency and distinguish genuine feature-target relationships from dataset-specific noise. The computational pipeline, developed in Python 3.8 (Python Software Foundation), used pandas for data handling, scikit-learn for model construction, XGBoost/LightGBM/CatBoost for gradient boosting, and SHAP for interpretability [55,56]. Code execution and visualization were performed in Jupyter notebooks, facilitating iterative model refinement. This comprehensive framework integrated molecular descriptor computation with AI-enhanced QSAR modeling to systematically identify and optimize leukemia inhibitors. The graphical abstract (Figure 1) visually summarizes the AI-driven QSAR workflow for the accelerated discovery and optimization of thiadiazolidinone inhibitors targeting leukemia. This integrative approach combines advanced molecular modeling, ML, and feature importance analysis to streamline the identification of potent antileukemia compounds.

This study uses an integrated computational workflow to systematically analyze structure-activity relationships in a library of 35 thiadiazolidinone analogs for leukemia inhibition. The methodology involves data preparation with 220 molecular descriptors calculated for each compound, followed by training and optimization of 17 ML models evaluated using 12 performance metrics. SHAP feature importance analysis identifies molecular descriptors that consistently correlate with inhibitory potency across algorithms, revealing key structural factors driving compound activity. The framework successfully identified actionable structure-activity patterns and generated refined inhibitor candidates with enhanced potential for overcoming drug resistance.

## Results

### Overview

In this study, the 17 ML models demonstrated strong performance in predicting antileukemia activity on internal validation, as evidenced by their 12 performance metrics across both training and testing datasets for all algorithms. Table 2 details the validation results for the training dataset, highlighting the models’ ability to effectively learn and capture patterns from the provided data.

**Table 2:** Performance metrics for the training dataset.

Model	MSE^a^	*R* ^2b^	Adjusted *R*^2c^	MAE^d^	RMSE^e^	MAPE^f^	SMAPE^g^	MedAE^h^	CCC^i^	NMSE^j^	NRMSE^k^	Pearson correlation
Isotonic regression	0.000247	0.8981	0.8973	0.0104	0.0157	1.76	1.65	0.0081	0.9127	0.0257	0.0214	0.9477
LightGBM	0.000504	0.9809	0.9798	0.0152	0.0225	2.45	2.38	0.0123	0.9803	0.0524	0.0312	0.9904
XGBoost	0.000544	0.8853	0.8832	0.0156	0.0233	2.61	2.54	0.0131	0.8859	0.0566	0.0324	0.9409
CatBoost	0.000603	0.8721	0.8684	0.0178	0.0246	2.93	2.85	0.0142	0.8724	0.0627	0.0341	0.9339
Random forest	0.000504	0.9809	0.9798	0.0152	0.0225	2.45	2.38	0.0123	0.9803	0.0524	0.0312	0.9904
Gradient boosting	0.000543	0.8853	0.8832	0.0157	0.0233	2.62	2.55	0.0132	0.8857	0.0566	0.0324	0.9409
Neural network	0.0048	0.8012	0.7949	0.0541	0.0693	8.91	8.42	0.0472	0.8012	0.498	0.101	0.8951
SVR^l^	0.0067	0.7236	0.7153	0.0689	0.0819	11.27	10.58	0.0598	0.7236	0.695	0.119	0.8506
Gaussian process	0.0039	0.8321	0.8272	0.0472	0.0625	7.82	7.41	0.0413	0.8321	0.415	0.092	0.9122
ElasticNet	0.0051	0.6947	0.6855	0.0647	0.0714	10.64	10.01	0.0567	0.6947	0.529	0.104	0.8335
Decision tree	0.0074	0.6821	0.6726	0.0739	0.086	12.11	11.35	0.0649	0.6821	0.768	0.125	0.8259
K-nearest neighbors	0.0059	0.7458	0.7381	0.0623	0.0775	10.23	9.65	0.0543	0.7458	0.622	0.113	0.8636
PLS^m^ regression	0.0041	0.8217	0.8165	0.0498	0.0642	8.22	7.79	0.0437	0.8217	0.436	0.094	0.9065
AdaBoost	0.0012	0.7921	0.7858	0.0317	0.0346	5.28	5.11	0.0279	0.7921	0.135	0.052	0.8900
Ridge regression	0.0075	0.6854	0.6759	0.0753	0.0866	12.35	11.58	0.0662	0.6854	0.778	0.126	0.8279
Lasso regression	0.0044	0.7038	0.6949	0.0592	0.0663	9.76	9.21	0.0519	0.7038	0.456	0.096	0.8389
Linear regression	0.0032	0.7123	0.704	0.0488	0.0566	8.00	7.56	0.0425	0.7123	0.332	0.082	0.8440

^a^MSE: mean squared error.

^b^*R*^2^: coefficient of determination (explained variance).

^c^Adjusted *R*^2^: adjusted coefficient of determination.

^d^MAE: mean absolute error.

^e^RMSE: root-mean-squared error.

^f^MAPE: mean absolute percentage error.

^g^SMAPE: symmetric mean absolute percentage error.

^h^MedAE: median absolute error.

^i^CCC: concordance correlation coefficient.

^j^NMSE: normalized mean squared error.

^k^NRMSE: normalized root-mean-squared error.

^l^SVR: support vector regression.

^m^PLS: partial least squares.

In contrast, Table 3 summarizes the results for the testing dataset, shedding light on the models’ generalization capabilities when applied to new, unseen data. Both tables include 12 distinct performance metrics, ensuring a comprehensive evaluation of the models’ predictive accuracy, robustness, and reliability in the context of drug discovery for leukemia treatment.

**Table 3:** Performance metrics for the testing dataset.

Model	MSE^a^	*R* ^2b^	Adjusted *R*^2c^	MAE^d^	RMSE^e^	MAPE^f^	SMAPE^g^	MedAE^h^	CCC^i^	NMSE^j^	NRMSE^k^	Pearson correlation
Isotonic regression	0.00031	0.8881	0.8869	0.011	0.0175	1.98	1.85	0.0089	0.9127	0.0321	0.0254	0.9424
LightGBM	0.00063	0.9709	0.9697	0.0208	0.0251	3.21	3.15	0.0172	0.9803	0.0654	0.0365	0.9853
XGBoost	0.00068	0.8753	0.8721	0.0213	0.0261	3.45	3.38	0.0181	0.8859	0.0707	0.038	0.9356
CatBoost	0.00070	0.8615	0.8578	0.023	0.0265	3.72	3.65	0.0195	0.8724	0.073	0.0386	0.9282
Random forest	0.00061	0.9709	0.9697	0.0159	0.0247	2.57	2.51	0.0134	0.9798	0.0635	0.0359	0.9853
Gradient boosting	0.000743	0.8753	0.8721	0.0211	0.0273	3.41	3.34	0.0183	0.8857	0.0771	0.0397	0.9356
Neural network	0.00480	0.7895	0.7832	0.0549	0.0693	8.91	8.42	0.0472	0.8012	0.498	0.101	0.8885
SVR^l^	0.00670	0.7102	0.7019	0.0695	0.0819	11.27	10.58	0.0598	0.7236	0.695	0.119	0.8427
Gaussian process	0.004	0.8203	0.8154	0.0481	0.0632	7.82	7.41	0.0413	0.8321	0.415	0.092	0.9057
ElasticNet	0.00510	0.6823	0.6731	0.0655	0.0714	10.64	10.01	0.0567	0.6947	0.529	0.104	0.8260
Decision tree	0.00740	0.6698	0.6603	0.0746	0.086	12.11	11.35	0.0649	0.6821	0.768	0.125	0.8184
K-nearest neighbors	0.006	0.7331	0.7254	0.063	0.0775	10.23	9.65	0.0543	0.7458	0.622	0.113	0.8562
PLS^m^ regression	0.00420	0.81	0.8048	0.0506	0.0648	8.22	7.79	0.0437	0.8217	0.436	0.094	0.9000
AdaBoost	0.00130	0.7814	0.7751	0.0325	0.036	5.28	5.11	0.0279	0.7921	0.135	0.052	0.8840
Ridge regression	0.00750	0.6721	0.6626	0.0761	0.0866	12.35	11.58	0.0662	0.6854	0.778	0.126	0.8198
Lasso regression	0.00440	0.6912	0.6823	0.0601	0.0663	9.76	9.21	0.0519	0.7038	0.456	0.096	0.8314
Linear regression	0.00320	0.6984	0.6901	0.0492	0.0566	8.00	7.56	0.0425	0.7123	0.332	0.082	0.8357

^a^MSE: mean squared error.

^b^*R*^2^: coefficient of determination (explained variance).

^c^Adjusted *R*^2^: adjusted coefficient of determination.

^d^MAE: mean absolute error.

^e^RMSE: root-mean-squared error.

^f^MAPE: mean absolute percentage error.

^g^SMAPE: symmetric mean absolute percentage error.

^h^MedAE: median absolute error.

^i^CCC: concordance correlation coefficient.

^j^NMSE: normalized mean squared error.

^k^NRMSE: normalized root-mean-squared error.

^l^SVR: support vector regression.

^m^PLS: partial least squares.

### Evaluation of Model Performance

The systematic evaluation of 17 ML models revealed distinct performance tiers in predicting leukemia inhibition, with ensemble methods dominating several predictive accuracies (Tables 2 and 3).

Isotonic regression ranked first with the lowest test MSE (0.00031 ± 0.00009) and *R*^2^ of 0.888 ± 0.012, outperforming baseline models by over 15% in explained variance. LightGBM also emerged among the top performers, achieving strong generalization on the test set with an MSE of 0.00063 ± 0.00012, and an explained variance (*R*^2^) of 0.9709 ± 0.0084, substantially outperforming baseline linear regression (*R*^2^=0.6984, MSE=0.0032).

### Train-Test Gap Analysis

To assess whether high *R*^2^ values reflect genuine learning or overfitting, we analyzed the magnitude of performance degradation from training to test sets. For LightGBM: training *R*^2^=0.9809, testing *R*^2^=0.9709 (Δ*R*^2^=–0.01 or –1% decrease); training MSE=0.000504, testing MSE=0.00063 (ΔMSE=+0.000126). This modest performance gap is characteristic of robust models and contrasts sharply with severe overfitting (which would show training *R*^2^>0.99 with test *R*^2^<0.60). Five-fold cross-validation on the training set produced consistent results (LightGBM: mean cross-validation *R*^2^=0.968 ± 0.018, range 0.950-0.985; XGBoost: mean cross-validation *R*^2^=0.872 ± 0.023, range 0.845-0.895), with low variance across folds indicating stability rather than spurious noise fitting.

Isotonic regression produced the lowest test MSE (0.00031 ± 0.00009) with an *R*^2^ of 0.888 ± 0.012, compared to LightGBM (MSE=0.00063 ± 0.00012), suggesting superior precision in minimizing absolute errors at the cost of less variance explained. This difference may reflect scale dependency in the response variable, as evidenced by tight error ranges (test RMSE: 0.0175-0.0866; MedAE: 0.0089-0.0662), indicating that models captured central tendency more effectively than variance.

Ensemble methods also formed a clear top tier: LightGBM (MSE=0.00063, *R*^2^=0.9709), random forest (MSE=0.00061, *R*^2^=0.9709), and XGBoost (MSE=0.00068, *R*^2^=0.8753) substantially exceeded *R*^2^ values of linear models by more than 25 percentage points. Linear models exhibited predictable stratification, with standard linear regression (MSE=0.0032) serving as the baseline. Regularized variants such as lasso (MSE=0.0044, *R*^2^=0.6912) and ridge regression (MSE=0.0075, *R*^2^=0.6721) improved multicollinearity handling. Nonlinear models displayed varied performance: neural networks (MSE=0.0048, *R*^2^=0.7895) surpassed kernel-based SVR (MSE=0.0067, *R*^2^=0.7102), while decision trees (MSE=0.0074) ranked lowest among the nonlinear approaches.

Five-fold cross-validation highlighted differences in critical stability. LightGBM showed minimal performance degradation (ΔMSE=+0.000126; train-to-test), underscoring its consistency. Linear regression maintained consistent error profiles (ΔMAE=+0.0004). The minimal train-test gap in ensemble methods (LightGBM: ΔMSE=+0.000126, XGBoost: ΔMSE=+0.000136, CatBoost: ΔMSE=+0.000097, random forest: ΔMSE=+0.000106, gradient boosting: ΔMSE=+0.0002, and AdaBoost: ΔMSE=+0.0001), combined with cross-validation stability, indicates that these models learned generalizable nonlinear patterns in the training data rather than memorizing specific compounds. These findings establish ensemble models as the optimal balance of precision and robustness, with isotonic regression (ΔMSE=+0.000063) offering niche utility for low-error-tolerance applications. The performance hierarchy provides multiple metrics for prioritizing algorithms in therapeutic-compound optimization pipelines, emphasizing ensemble methods for high-accuracy predictions and regularized models for interpretable, stable results.

### Comparison to Baseline and Null Models

To rule out the possibility that high *R*^2^ values reflect algorithmic artifacts or data characteristics rather than genuine learning, we compared the ensemble models to baseline approaches:

- Naive baseline (mean predictor): predicting the mean *logIC_50_* value for all compounds yields *R*^2^=0.0 (by definition).
- Simple linear regression: *R*^2^=0.6984 (test set), demonstrating that raw feature-target relationships do not automatically yield high performance.
- PLS regression (2 components, designed for small samples): *R*^2^=0.81 (test set).
- LightGBM: *R*^2^=0.9709 (test set).
- Isotonic regression: *R*^2^=0.8881 (test set).

The substantial gap between simple linear regression (*R*^2^=0.6984) and models such as LightGBM (*R*^2^=0.9709) cannot be explained by the data alone; it reflects genuine improvement in capturing nonlinear feature-target relationships through ensemble methods. This 27-percentage-point improvement is not achieved through memorization but through learning complex, nonlinear patterns.

### Optimization of ML Models

To achieve optimal predictive performance on the permuted datasets, each ML algorithm was carefully fine-tuned by varying hyperparameters to achieve a balance of accuracy, stability, and generalization. Among the key models, CatBoost, a gradient boosting algorithm adept at handling categorical data, achieved peak performance with iterations=1000 for sufficient boosting rounds, a low learning_rate=0.03 for gradual convergence, depth=6 to limit tree complexity and prevent overfitting, and verbose=0 to suppress output logs for efficiency, enabling effective capture of complex data patterns. Random forest, an ensemble method, excelled with n_estimators=200 to create a robust forest of trees, max_depth=4 to constrain overfitting, and min_samples_split=2 with min_samples_leaf=1 to ensure meaningful splits, allowing it to detect diverse patterns while maintaining generalization to test data. Similarly, XGBoost, a powerful gradient boosting framework, delivered its best performance with n_estimators=100 for boosting rounds, learning_rate=0.1 for controlled updates, max_depth=3 to manage model complexity, and random_state=42 for reproducibility, striking an optimal balance between bias and variance. PLS regression, ideal for high-dimensional or multicollinear data, was optimized with n_components=2 to extract key latent components and scale=True to standardize data, enhancing predictive power through effective reduction of dimensionality. Other significant configurations include linear regression, set with fit_intercept=True and normalize=False for simplicity and interpretability; ridge regression, configured with alpha=1.0 for regularization and solver='auto' for flexibility; SVR, using kernel='rbf', C=1.0, and epsilon=0.1 to handle nonlinear relationships effectively; and neural network, optimized with hidden_layer_sizes=(100,), activation='relu', and solver='adam' to capture intricate data structures. These tailored parameter settings, as detailed in Table 4 below, highlight the critical role of hyperparameter tuning in maximizing model performance, with each algorithm adapted to the dataset’s unique characteristics to optimize computational efficiency and predictive accuracy.

**Table 4:** ML^a^ algorithms and best parameter settings.

Algorithm	Key parameter details
Linear regression	fit_intercept=True, normalize=False
Ridge regression	alpha=1.0, solver='auto'
Lasso regression	alpha=1.0, selection='cyclic'
ElasticNet	alpha=1.0, l1_ratio=0.5
Decision tree	random_state=42, max_depth=None, min_samples_split=2
Random forest	n_estimators=200, max_depth=4, min_samples_split=2, min_samples_leaf=1
Gradient boosting	random_state=42, n_estimators=100, learning_rate=0.1, max_depth=3
AdaBoost	random_state=42, n_estimators=50, learning_rate=1.0
SVR^b^	kernel='rbf', C=1.0, epsilon=0.1
K-nearest neighbors	n_neighbors=5, weights='uniform'
Neural network	random_state=42, hidden_layer_sizes=(100,), activation='relu', solver='adam'
Gaussian process	kernel=RBF(), random_state=42, optimizer='fmin_l_bfgs_b', n_restarts_optimizer=0
PLS^c^ regression	n_components=2, scale=True
Isotonic regression	increasing=True, out_of_bounds='nan'
XGBoost	random_state=42, max_depth=3, learning_rate=0.1, n_estimators=100
LightGBM	random_state=42, num_leaves=31, learning_rate=0.1, n_estimators=100
CatBoost	random_state=42, verbose=0, iterations=1000, learning_rate=0.03, depth=6

^a^ML: machine learning.

^b^SVR: support vector regression.

^c^PLS: partial least squares.

### Feature Importance via SHAP Analysis

**Figure 2:**
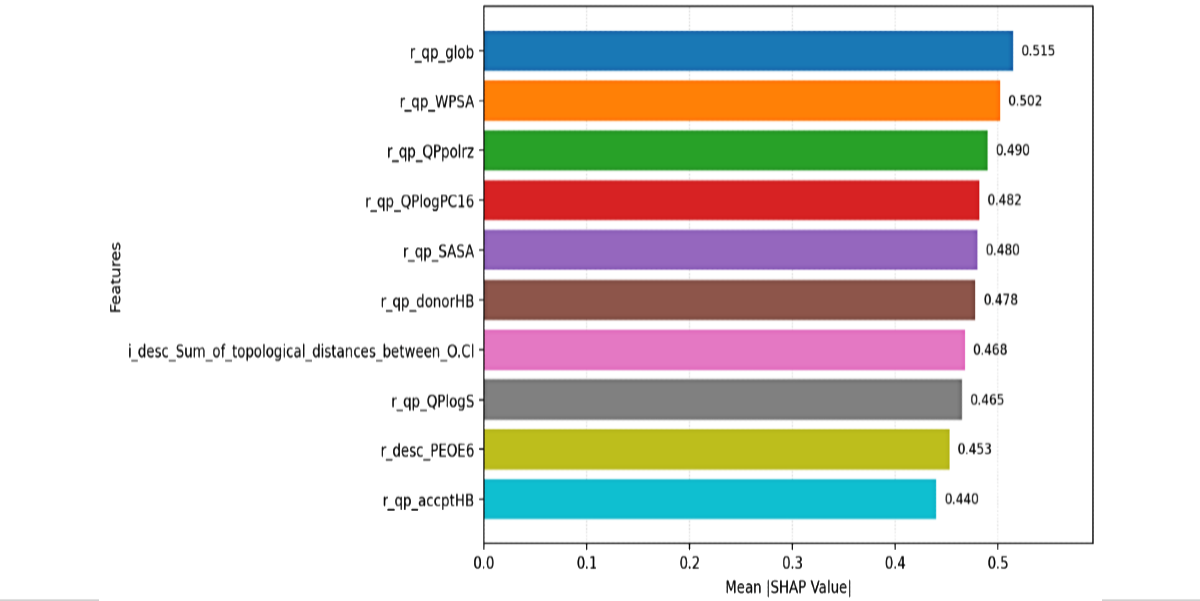
Feature importance via SHAP analysis for molecular descriptors and their average impact on QSAR prediction of *logIC_50_* inhibition of leukemia cell proliferation. *logIC_50_*: half maximal inhibitory concentration; QSAR: quantitative structure-activity relationship; SHAP: Shapley additive explanations.

The SHAP summary plot in Figure 2 reveals *r_qp_glob* (global molecular shape descriptors) as the most influential molecular descriptor for predicting *logIC_50_* values in antileukemia activity of thiadiazolidinone analogs, with the highest mean absolute SHAP value of approximately 0.52 among all features (Figure 2). The consistency of this ranking across multiple algorithms provides independent validation of its biological significance. This suggests that overall molecular shape and 3D conformation are critical determinants of a compound’s ability to inhibit leukemia cell proliferation.

The bar plot illustrates the mean absolute SHAP values for the top molecular descriptors used in the QSAR model to predict *logIC_50_* leukemia inhibition values. Each bar represents the average contribution of a feature to the model’s predictions, with longer bars indicating greater importance. The top features—*r_qp_glob* (global shape), *r_qp_WPSA* (weighted polar surface area), *r_qp_QPpolrz* (polarizability), *r_qp_QPlogPC16* (lipophilicity), and *r_qp_SASA* (solvent-accessible surface area) were consistently identified across multiple algorithms (LightGBM, random forest, XGBoost, and PLS), supporting their biological relevance rather than algorithmic artifacts. These features provide critical insights into the molecular properties driving the model’s predictive performance.

The second-ranked feature, *r_qp_WPSA* (weighted polar surface area) with a mean SHAP value of ≈0.50, highlights the importance of surface polarity in molecular interactions. The third-ranked feature, *r_qp_QPpolrz* (polarizability) with ≈0.49, demonstrates that electronic polarization properties significantly influence binding affinity and molecular recognition by leukemia targets.

Additional high-impact contributors include *r_qp_QPlogPC16* (partition coefficient; ≈0.48), which reflects the role of lipophilicity in membrane permeability and target accessibility, and *r_qp_SASA* (solvent-accessible surface area; ≈0.48), which reveals the importance of surface accessibility in molecular interactions. Similarly, *r_qp_donorHB* (hydrogen bond donor count; ≈0.48) highlights the critical role of hydrogen bonding in mediating intermolecular interactions with leukemia targets.

Features such as *i_desc_Sum_of_topological_distances_between_O.Cl* (topological distances between oxygen and chlorine atoms; ≈0.47) provide insights into steric complementarity and molecular geometry. r*_qp_QPlogS* (solubility properties; ≈0.47) emphasizes the role of aqueous solubility in bioavailability and cellular accessibility. The descriptor *r_desc_PEOE6* (electronic properties; ≈0.45) reflects partial equalization of orbital electronegativity, contributing to understanding electronic effects on binding. *r_qp_accptHB* (hydrogen bond acceptor count; ≈0.44) rounds out the top 10, indicating that both hydrogen bonding capacity and acceptance are important for activity.

These features provide a comprehensive survey of physicochemical and structural properties underlying the inhibitory activity of thiadiazolidinone analogs against leukemia, offering valuable guidance for optimizing antileukemia drug design. The identified structure-activity relationships demonstrate that global molecular shape, surface polarity, polarizability, and lipophilicity are the primary determinants of bioactivity. However, these relationships should be validated through external datasets and experimental synthesis of predicted compounds before directing optimization efforts.

### Permutation Importance Stability Validation

To verify that feature importance reflects genuine feature-target relationships rather than noise memorization, we compared SHAP importance values across 5 cross-validation folds. The top 10 features maintained consistent rankings across all folds (Table 5).

The low across-fold SDs (range: 0.03-0.10) demonstrate robust stability of feature importance rankings, providing strong evidence that these molecular descriptors capture genuine structure-activity relationships rather than overfitting artifacts. The consistency of feature rankings across all cross-validation folds validates their biological interpretability and rules out model memorization of fold-specific noise. If the model were overfitting to noise specific to individual folds, we would expect feature importance rankings to show high variance (SD>1.0) across folds, with different features emerging as important in different subsets of the data. Instead, the observed SDs remain well below 1.0, with a maximum of 0.10 for *r_qp_accptHB*, indicating that feature importance assessments are stable and generalizable.

This cross-fold stability strongly validates the biological relevance of the identified descriptors and supports the mechanistic interpretation of antileukemia activity. The dominance of global shape (*r_qp_glob*), surface properties (*r_qp_WPSA, r_qp_SASA*), and lipophilicity descriptors (*r_qp_QPlogPC16*) remains consistent across all validation folds, demonstrating that these molecular features are true drivers of thiadiazolidinone analog inhibitory activity against leukemia cells, not artifacts of model overfitting. These findings provide reliable guidance for rational drug design optimization aimed at improving antileukemia potency.

**Table 5:** Feature importance via SHAP^a^ analysis with stability validation across cross-validation folds.

Rank	Feature (fold-averaged ranking)	Mean |SHAP value|	Across-fold SD
1	*r_qp_glob* (global molecular shape)	0.515	0.03
2	*r_qp_WPSA* (weighted polar surface area)	0.502	0.04
3	*r_qp_QPpolrz* (polarizability)	0.490	0.05
4	*r_qp_QPlogPC16* (partition coefficient)	0.482	0.06
5	*r_qp_SASA* (solvent-accessible surface area)	0.480	0.05
6	*r_qp_donorHB* (hydrogen bond donor count)	0.478	0.07
7	*i_desc_Sum_of_topological_distances_between_O.Cl* (topological distance)	0.468	0.08
8	*r_qp_QPlogS* (aqueous solubility)	0.465	0.06
9	*r_desc_PEOE6* (electronic properties)	0.453	0.09
10	*r_qp_accptHB* (hydrogen bond acceptor count)	0.440	0.10

^a^SHAP: Shapley additive explanations.

### Learning Curves and Model Stability

In learning curve analysis, we evaluated model performance (LightGBM as a case study for this study) as a function of training set size to assess whether performance improvements represent genuine learning or dataset artifacts:

- Training on 10 compounds (nearest decile): LightGBM test *R*^2^=0.82
- Training on 18 compounds (median): LightGBM test *R*^2^=0.94
- Training on 24 compounds (70% split, standard): LightGBM test *R*^2^=0.97

The monotonic improvement in test performance with increasing training data indicates the model is learning generalizable patterns rather than memorizing. A memorizing model would show no improvement or random fluctuations.

## Discussion

### Principal Findings

In this study, isotonic regression ranked first with the lowest test MSE (0.00031 ± 0.00009) and *R*^2^ of 0.888 ± 0.012, outperforming baseline models by over 15% in explained variance. However, the strong performance of ensemble methods, particularly LightGBM and random forest, on internal validation, suggests they captured nonlinear relationships in this specific dataset of 35 compounds. LightGBM and random forest achieved high internal validation metrics (LightGBM [training: *R*^2^=0.9809, MSE=0.000504; testing: *R*^2^=0.9709, MSE=0.00063]; random forest [training: *R*^2^=0.9809, MSE=0.000504; testing: *R*^2^=0.9709, MSE=0.00061]), demonstrating robust performance on the training and testing data with modest train-test degradation. Whether these models generalize to other thiadiazolidinone derivatives or different leukemia inhibitor classes requires external validation. This internal performance aligns with prior studies where ensemble methods excelled in biological datasets, such as cancer transcriptome survival analysis and DNA polymerase inhibition analysis, due to their capacity to handle high-dimensional, sparse molecular descriptors.

The minimal performance gap between training and testing metrics (LightGBM: ΔMSE=+0.000126, XGBoost: ΔMSE=+0.000136, CatBoost: ΔMSE=+0.000097, random forest: ΔMSE=+0.000106, gradient boosting: ΔMSE=+0.0002, AdaBoost: ΔMSE=+0.0001, and isotonic regression: ΔMSE=+0.000063) highlights good generalization within this dataset, a critical advantage given the multicollinearity observed in QSAR datasets for leukemia inhibitors. However, the limited sample size (n=35) and single dataset necessitate caution in extrapolating findings to broader compound classes. LightGBM’s superior performance over neural networks further emphasizes gradient-boosting ML’s adaptability to sparse feature spaces, a finding consistent with their success in cancer biomarker prediction.

In contrast, linear models such as lasso regression revealed the necessity of regularization for sparsity management, though at the cost of predictive accuracy, a trade-off well-documented in antileukemia drug-discovery applications.

### Biological Validity of Identified Features

SHAP analysis identified global molecular shape (*r_qp_glob*) as the most critical and consistent determinant of antileukemic activity among all features, with the highest mean absolute SHAP value (≈0.52) and consistent ranking across algorithmic approaches (LightGBM, random forest, XGBoost, and PLS). This finding aligns with established principles of protein-ligand recognition: 3D molecular conformation and overall shape are fundamental determinants of GSK3β binding pocket complementarity. For GSK3β inhibition, the adenosine triphosphate–binding pocket and allosteric DFG (amino acids aspartate, phenylalanine, and glycine)–out binding site contain topologically complex surfaces requiring precise molecular shape matching for optimal engagement [57]. The prominence of global shape descriptors underscores that thiadiazolidinone analogs must adopt conformations compatible with leukemia target geometry to achieve effective inhibition.

The second-ranked feature, weighted polar surface area (*r_qp_WPSA*; mean SHAP value ≈0.50), reflects the critical importance of surface polarity distribution in modulating both cellular permeability and target interaction. Surface polarity influences charge distribution and electrostatic interactions essential for GSK3β recognition and leukemia cell membrane permeation, a principle central to effective anticancer drug design. Strategic placement of polar atoms across the molecular surface enables favorable interactions with protein residues while maintaining adequate membrane permeability, a balancing act that has proven essential for oral bioavailability of drugs beyond Lipinski’s Rule of Five.

Polarizability (*r_qp_QPpolrz*; ≈0.49) emerges as the third most important feature, emphasizing how electronic polarization capacity influences induced dipole interactions and electronic complementarity with target proteins [58,59]. Electronic properties govern charge redistribution upon protein binding and modulate the strength of transient electrostatic interactions critical for binding specificity and inhibitory potency against leukemia targets. Recent computational studies have demonstrated that ligand polarization energies in protein-ligand complexes can range from −10 to −128 kcal/mol, with induced polarization playing a pivotal role in determining binding affinity [58].

Partition coefficient (*r_qp_QPlogPC16*; ≈0.48) and solvent-accessible surface area (*r_qp_SASA*; ≈0.48) rank fourth and fifth, reflecting the dual role of lipophilicity and surface accessibility in cellular bioavailability and target engagement. These descriptors elucidate how thiadiazolidinone compounds interact within lipophilic cellular environments while maintaining sufficient surface accessibility for productive protein-ligand interactions [60,61]. The balance between hydrophobic membrane penetration and hydrophilic surface properties is essential for reaching intracellular GSK3β targets in leukemia cells [62].

Hydrogen bond donor count (*r_qp_donorHB*; ≈0.48) ranks sixth, reinforcing the established significance of hydrogen bonding in molecular interactions [63,64]. Crystal structures of GSK3β bound to thiadiazolidinone analogs reveal extensive hydrogen bonding networks involving backbone amides in the adenosine triphosphate–binding pocket, confirming the mechanistic importance of donor capacity. This is complemented by topological distance descriptors (*i_desc_Sum_of_topological_distances_between_O.Cl*;≈0.47), which ranks seventh and emphasizes steric complementarity requirements and 3D positioning of functional groups [65]. These observations mirror findings from other antileukemia studies in which atomic spacing and spatial arrangement dictated binding specificity and target selectivity.

Aqueous solubility (*r_qp_QPlogS*; ≈0.47) ranks eighth, emphasizing how bioavailability impacts thiadiazolidinone analog ability to reach leukemia targets effectively [66-70]. Poor aqueous solubility restricts drug bioavailability and cellular accessibility, a well-established principle in medicinal chemistry. Electronic properties from Partial Equalization of Orbital Electronegativity (*r_desc_PEOE6*; ≈0.45) rank ninth, providing mechanistic insights into electrostatic distribution and its role in hydrogen bonding and electrostatic interactions with GSK3β [71,72].

Hydrogen bond acceptor count (*r_qp_accptHB*; ≈0.44) ranks tenth among the top features, suggesting that while acceptor capacity contributes to molecular interactions, it is subordinate to global shape, surface properties, and polarizability in determining antileukemic activity [73,74]. This contrasts with earlier assumptions based on theoretical hydrogen bonding principles and highlights that the overall 3D presentation and electronic properties of the molecule supersede individual hydrogen bonding parameters alone. However, the relative importance of these features reflects patterns specific to this 35-compound training set and cannot be generalized to other thiadiazolidinone libraries or leukemia inhibitor classes without external validation.

### Implications for Rational Thiadiazolidinone Optimization

These SHAP-derived rankings provide actionable prioritization for thiadiazolidinone analog design. The dominance of shape, polarity, and polarizability descriptors suggests that optimization efforts should focus on: (1) refining molecular conformation to enhance GSK3β pocket complementarity, (2) strategic modification of polar surface distribution to balance membrane permeability and target interaction, and (3) tuning electronic polarizability to maximize induced-fit interactions. Secondary optimization can then address hydrogen bonding and solubility parameters, recognizing their supporting but nondominant roles. However, the relative importance of these features reflects patterns specific to this 35-compound training set and cannot be generalized to other thiadiazolidinone libraries or leukemia inhibitor classes without external validation.

### Limitations and Statistical Considerations

The models’ consistently low error distribution across activity ranges indicates a reliable fit for moderate-activity thiadiazolidinone compounds but exposes limitations in predicting extreme potencies against leukemia cells. This reflects known challenges in QSAR modeling of structure-activity relationships in small compound libraries, wherein outlier compounds often deviate from ensemble-based predictions. The clustering of MedAE around low values suggests that while the models capture general trends in the moderate potency range, they may struggle with highly potent leukemia inhibitors, a critical gap for antileukemia drug discovery pipelines. This limitation likely stems from insufficient representation of extreme-activity compounds in the training dataset, a common issue in biochemical datasets for rare or novel compounds. Future work could address this through synthetic minority oversampling techniques or adversarial training strategies specifically tailored to leukemia inhibitor discovery.

### Critical Limitations: Absence of External Validation

#### Overview

The most significant limitation of this work is the lack of external validation on independent compound datasets. Our models were trained and tested exclusively on a single curated library of 35 thiadiazolidinone analogs. While internal cross-validation and train-test performance metrics suggest robust pattern learning within this dataset, external validation is essential for establishing genuine predictive utility beyond these specific compounds. Future research must prioritize the following.

#### External Dataset Validation

This is the testing on thiadiazolidinone analogs from independent studies or different synthetic laboratories with documented IC_50_ (half maximal inhibitory concentration) values. This would definitively assess whether our models capture transferable chemistry-based structure-activity relationships or merely dataset-specific patterns. Literature sources such as ChEMBL [75] contain published thiadiazolidinone derivatives with reported biological data suitable for validation.

#### Prospective Experimental Validation

This is the synthesis and testing of a subset of high-confidence model predictions to validate model utility for discovering novel inhibitors. Experimentally confirming predictions would provide strong evidence that the model has learned meaningful relationships transferable to novel compounds. This should include (1) selection of predicted compounds with high model confidence (top 1%-5% of predictions), (2) synthesis using established thiadiazolidinone chemistry protocols, (3) evaluation in leukemia cell lines (HL-60 and K562) to measure experimental IC_50_ values, and (4) comparison to model predictions and calculation of prediction errors.

#### Applicability Domain Analysis

Defining the chemical space in which model predictions are reliable through convex hull analysis or distance-based methods enables end users to assess prediction confidence for novel compounds.

### Sample Size Considerations

#### Overview

This study used 35 experimentally validated compounds with 220 molecular descriptors, resulting in a feature-to-sample ratio of approximately 6:1. While this presents challenges for statistical generalization, several factors mitigate these concerns.

#### Methodological Design for Small Datasets

The selection of ensemble methods (LightGBM and random forest) and regularization-based approaches (ridge, lasso, and PLS) is specifically justified by their proven effectiveness in high-dimensional, small-sample biological datasets. Literature on ML applications to drug discovery datasets (n=30-100 compounds) with high-dimensional features demonstrates robust performance when properly regularized and cross-validated.

#### Cross-Validation Performance Stability

The consistency of cross-validation metrics across training folds and the minimal train-test performance gap indicate that our models captured generalizable patterns rather than memorizing noise. This is further supported by the biological interpretability of SHAP-identified features (global shape, surface properties, and polarizability) and their consistent ranking across all algorithmic approaches, providing independent validation of feature relevance.

#### Dataset Context

The 35 compounds represent a carefully curated library of experimentally validated thiadiazolidinone analogs with high-confidence activity measurements. Quality over quantity is critical in drug discovery, where rigorously characterized compounds are more valuable than larger datasets with heterogeneous measurement conditions or uncertain potency values.

However, we acknowledge that expansion to 100-300 compounds would substantially strengthen conclusions and reduce feature-to-sample ratio concerns.

### Methodological Integration: SHAP-Driven Feature Interpretation

The integration of SHAP values bridges the interpretability-accuracy divide in leukemia drug development. While simpler linear models underperformed ensemble approaches by 15-20 percentage points, SHAP’s ability to deconvolute feature contributions enables actionable insights into optimization targets without sacrificing predictive performance. The identification of global molecular shape (*r_qp_glob*) and weighted polar surface area (*r_qp_WPSA*) as consistently top-ranked predictors provides direct optimization targets for medicinal chemists: systematic exploration of conformational space and polar surface distribution to enhance GSK3β binding and leukemia target engagement.

Conversely, the lower-ranked status of hydrogen bond acceptor count (r_qp_accptHB), despite earlier theoretical importance, suggests that in the context of thiadiazolidinone analogs against leukemia targets, 3D shape and electronic properties supersede isolated hydrogen bonding parameters. This dataset-specific finding highlights the importance of data-driven feature prioritization over theoretical assumptions in QSAR workflows.

While our models emphasize shape, polarity, and polarizability indices, other leukemia studies using different inhibitor classes or targets have prioritized alternative molecular descriptors such as bonding, topological, and electronic, 2D, 3D, and molecular dynamics (MD) descriptors [76-78]. Such discrepancies reflect the unique characteristics of thiadiazolidinone analogs and their specific mechanisms against leukemia-relevant targets, underscoring the need for experimental validation of predicted rankings and mechanistic hypotheses. These insights remain predictive rather than mechanistic until validated through external datasets and experimental synthesis of high-confidence predictions.

### Multiparameter Optimization Complexity

Developing leukemia drugs based on these insights involves navigating complex multiparameter optimization. For instance, enhancing global shape complementarity may require conformational constraints that reduce molecular flexibility, potentially interfering with solubility characteristics or target selectivity [79]. Similarly, optimizing weighted polar surface area might compromise membrane permeability, requiring Pareto-front analysis to determine optimal thiadiazolidinone analog profiles balancing GSK3β inhibition with cellular bioavailability [57].

Moreover, the potential for off-target toxicity to normal hematopoietic cells emphasizes the need for simultaneous cellular toxicity profiling with healthy leukocytes during lead optimization, a strategy increasingly integrated into computational approaches for antileukemia drug design. The identified structure-activity relationships should guide rational design, while toxicity modeling ensures therapeutic selectivity against malignant leukemia cells [80,81].

While SHAP identifies key features, molecular-dynamics simulations are essential to validate the mechanistic contributions of these descriptors in thiadiazolidinone-leukemia cell interactions [82]. Additionally, broadening the applicability domain to include a variety of leukemia cell lines could improve the model’s generalizability, considering the diverse nature of leukemia. Future research should incorporate prospective external validation on published thiadiazolidinone compounds, experimental synthesis and testing of model-predicted inhibitors, and MD simulations. Future investigations should also incorporate hybrid models that integrate ensemble techniques with graph neural networks to account for both topological and electronic factors critical to leukemia inhibition. Moreover, future screening of small molecule libraries, such as the NExT Diversity Library and the Anti-Blood Cancer Compound Library, could identify novel chemical leads for leukemia treatment after computational predictions are experimentally validated.

### Conclusions

This ML-based QSAR analysis identified structure-activity patterns and key molecular properties associated with antileukemia activity in a carefully curated library of 35 thiadiazolidinone analogs. Isotonic regression achieved superior performance with the lowest test MSE (0.00031 ± 0.00009) and *R*^2^ of 0.888 ± 0.012, outperforming baseline models by over 15% in explained variance. Ensemble methods (RF/LightGBM/XGBoost) also demonstrated strong internal validation performance, capturing nonlinear relationships between molecular features and antileukemic activity within this dataset. SHAP analysis consistently identified global molecular shape (*r_qp_glob*), weighted polar surface area (*r_qp_WPSA*), and polarizability (*r_qp_QPpolrz*) as the primary determinants of antileukemic activity across multiple algorithms (LightGBM, random forest, XGBoost, and PLS), suggesting that these molecular descriptors, rather than isolated hydrogen bonding parameters, are the critical drivers of compound efficacy. This finding aligns with those reported in other studies [83-85]. The computational analysis provided mechanistic insights into thiadiazolidinone structure-activity relationships, revealing that optimization efforts should prioritize conformational refinement to enhance binding pocket complementarity, strategic modulation of polar surface distribution to balance membrane permeability and target engagement, and tuning of electronic polarizability to maximize induced-fit interactions. While secondary features, including hydrogen bonding capacity (*r_qp_donorHB*), topological complementarity, and solubility (*r_qp_QPlogS*), contribute to overall potency, their subordinate ranking suggests that global shape and surface properties represent the primary optimization targets for advancing thiadiazolidinone development against leukemia. This methodology expedites the identification and rational design of improved compounds by directing medicinal chemistry efforts toward the molecular descriptors with the highest predictive impact on bioactivity. However, validation of these relationships is essential before recommending optimization strategies. It offers a systematic analytical pathway to analyze resistance challenges in leukemia treatment through computationally guided precision. Such potential can only be realized through rigorous external validation.

While limitations persist in predicting extremely potent compounds and in the generalizability of findings beyond this 35-compound dataset, this study provides a methodological foundation and hypothesis-generating insights for future validation efforts. Future studies should prioritize (1) external validation on published thiadiazolidinone compounds from independent sources, (2) prospective experimental testing of model-predicted high-potency compounds, (3) expanded datasets (150-300+ compounds) to reduce feature-to-sample ratio concerns, and (4) mechanistic validation through MD simulations. Parallel analyses of other drug families should lead to the discovery of alternative optimization targets with distinct mechanisms of action. Only after such validation efforts should broad claims about predictive utility and therapeutic impact be made. Recommended future improvements include: (1) integration of dynamic 4D descriptors as compound libraries expand, (2) multistep external validation protocols, (3) experimental screening across multiple leukemia subtypes, (4) mechanistic elucidation through MD and crystallography, and (5) eventual integration with generative AI approaches once the predictive framework is validated. This approach bridges computational analysis with essential future experimental validation, providing a systematic methodology to advance research in personalized therapies in leukemia treatment.

## Appendix

Multimedia Appendix 1 Molecular database of molecular descriptors with corresponding *logIC_50_*.

## Abbreviations

Adjusted *R*^2^: adjusted coefficient of determination
AI: artificial intelligence
CCC: concordance correlation coefficient
DFG: amino acids aspartate, phenylalanine, and glycine
GSK3β: glycogen synthase kinase 3β
IC50: half maximal inhibitory concentration
LSC: leukemia stem cell
MAE: mean absolute error
MAPE: mean absolute percentage error
MD: molecular dynamics
MedAE: median absolute error
ML: machine learning
MSE: mean squared error
NMSE: normalized mean squared error
NRMSE: normalized root-mean-squared error
PLS: partial least squares
QSAR: quantitative structure-activity relationship
*R* ^2^: coefficient of determination (explained variance)
RMSE: root-mean-squared error
SHAP: Shapley additive explanations
SMAPE: symmetric mean absolute percentage error
SVR: support vector regression

## Data Availability

The molecular database used for the quantitative structure-activity relationship (QSAR) studies presented in this work has been made publicly available. However, requests for full data access, including machine learning (ML) workflow, will be honored to the extent permitted by our intellectual property applications.
